# Systemic immune-inflammation index and systemic inflammation response index: novel hematological predictors of obstructive sleep apnea

**DOI:** 10.3389/fneur.2026.1716598

**Published:** 2026-03-12

**Authors:** Guohua He, Mingzhu Deng, Yangping Tong, Zhen Wan, Wei Xu, Tieqiao Feng, Wengao Zeng, Ling Xiao, Dandan Yang, Kangping Song, Fangyi Li

**Affiliations:** 1 Department of Neurology, The Affiliated Changsha Central Hospital, Hengyang Medical School, University of South China, Changsha, Hunan, China; 2 Department of Neurology, The Second People’s Hospital of Hunan Province (Brain Hospital of Hunan Province), Changsha, Hunan, China

**Keywords:** apnea-hypopnea index, inflammation, obstructive sleep apnea, systemic immune-inflammatory index, systemic inflammation response index

## Abstract

**Background:**

While the association between obstructive sleep apnea (OSA) and chronic inflammation is well-established, the role of novel hematological indices, such as the systemic immune-inflammation index (SII) and systemic inflammation response index (SIRI), remains unclear. Their specific relationship with OSA has not been fully elucidated.

**Methods:**

A total of 590 consecutive patients with suspected OSA were enrolled between June 2023 and August 2025. OSA was defined as an apnea-hypopnea index (AHI) of ≥5 events per hour. Spearman’s rank correlation analysis was used to assess the relationships among SII, SIRI, AHI, and the lowest SpO₂. Logistic regression analyses were performed to examine the associations of SII and SIRI with OSA. Sensitivity analyses were conducted to evaluate the robustness of the findings. Receiver operating characteristic (ROC) curves were generated to assess the discriminative ability of SII and SIRI in identifying patients with OSA.

**Results:**

Of the 590 patients included in the study, 413 (70%) were diagnosed with OSA. Spearman rank correlation analysis revealed that both SII (*r* = 0.541, *p* < 0.001) and SIRI (*r* = 0.412, *p* < 0.001) were positively correlated with AHI. Conversely, SII (*r* = −0.470, *p* < 0.001) and SIRI (*r* = −0.374, *p* < 0.001) were inversely correlated with the lowest SpO₂. Binary logistic regression identified both SII (odds ratio [OR] = 1.601; 95% CI: 1.352–1.987; *p* < 0.001) and SIRI (OR = 1.459; 95% CI: 1.182–1.750; *p* < 0.001) as independent risk factors for OSA. The area under the curve (AUC) values for predicting OSA were 0.774 for SII, 0.705 for SIRI, and 0.819 for the combination of SII and SIRI.

**Conclusion:**

Our findings demonstrate that both the SII and SIRI are independent risk factors for OSA and may serve as potential biomarkers for identifying individuals with OSA.

## Introduction

Obstructive sleep apnea (OSA) is a sleep disorder characterized by recurrent intermittent collapse of the upper airway, resulting in periodic hypoxemia and sleep fragmentation (1). Substantial evidence supports a strong association between OSA and an increased risk of cardiovascular diseases (2–4). With an estimated prevalence ranging from 9 to 38% in the general population (5), OSA represents a considerable public health burden. Untreated OSA significantly elevates the risk of cardiovascular and cerebrovascular events (6) and is associated with approximately $150 billion in annual healthcare-related costs (7). However, OSA remains underrecognized, posing challenges for its prevention and management (8). Although polysomnography is the current gold standard for diagnosing OSA (9), home sleep apnea testing offers an alternative with approximately 80% sensitivity (1). Nevertheless, limited access to diagnostic equipment in many settings underscores the need to identify readily measurable biomarkers and risk factors for OSA.

Sleep and circadian rhythm fluctuations dynamically regulate immune function through mechanisms such as immune cell redistribution and inflammatory mediator production (10). Sleep disturbances, which are implicated in a wide range of pathologies, are known to disrupt immune homeostasis, leading to excessive inflammatory activation that may accelerate disease progression (11, 12). These interactions are considered bidirectional (13). Systemic inflammation—whether triggered by endogenous factors or infections—can feedback to disrupt normal sleep architecture. Pro-inflammatory cytokines, such as interleukin-1β (IL-1β), interleukin-6 (IL-6), and tumor necrosis factor-α (TNF-α), can influence central nervous system functions via both neural and humoral pathways after crossing the blood–brain barrier, promoting sleepiness, altering sleep-stage distribution (e.g., increasing non-rapid eye movement sleep), and fragmenting sleep (10, 13, 14). OSA, a multisystem disorder, is strongly linked to chronic systemic inflammation, which represents a core pathophysiological mechanism. Elevated levels of circulating inflammatory markers have been consistently observed in patients with OSA, it is hypothesized that intermittent hypoxemia resulting from apnea-hypopnea episodes activates multiple inflammatory pathways (15). In recent years, two integrated inflammatory indices have gained attention: the systemic immune-inflammatory index (SII) and the systemic inflammatory response index (SIRI) (16). Elevated SII has been associated with poorer outcomes in conditions including solid tumors, pulmonary embolism, cardiovascular diseases, and COVID-19 (17–19). It may also serve as a predictor of early neurological deterioration and poor 90-day functional outcomes following intravenous thrombolysis (20). Some studies suggest that SII reflects chronic inflammation in OSA and could represent a readily measurable, cost-effective biomarker (21, 22). Furthermore, sleep disturbances, OSA symptoms, and daytime sleepiness have been positively correlated with SII in U. S. adults (23). Nonetheless, the association between SII and OSA remains contentious. A significant correlation between AHI and SII has been reported only in severe OSA subgroups (24), and a recent study found no link between SII and OSA severity (25). Thus, the role of SII in OSA warrants further investigation. The SIRI is a novel composite index used to reflect systemic inflammation and immune response. Similarly, SIRI has emerged as a composite marker reflecting systemic inflammation and immune status. It has been utilized to predict prognosis in pneumonia, rheumatoid arthritis, and acute pancreatitis (26–28), and has demonstrated high predictive value for cardiovascular events (29–32). Despite growing evidence supporting the clinical relevance of SIRI, its relationship with OSA has not been established.

Numerous risk factors—including male sex, elevated body-mass index, asthma, the rs12415421 genetic polymorphism, and insulin resistance/hyperglycemia—have been established as associated with OSA (33). However, the relationship between novel inflammatory indices, namely the SII and SIRI, and OSA remains incompletely understood. Therefore, this study aimed to evaluate the association of SII and SIRI with the risk of OSA.

## Methods study design and subjects

**Figure 1:**
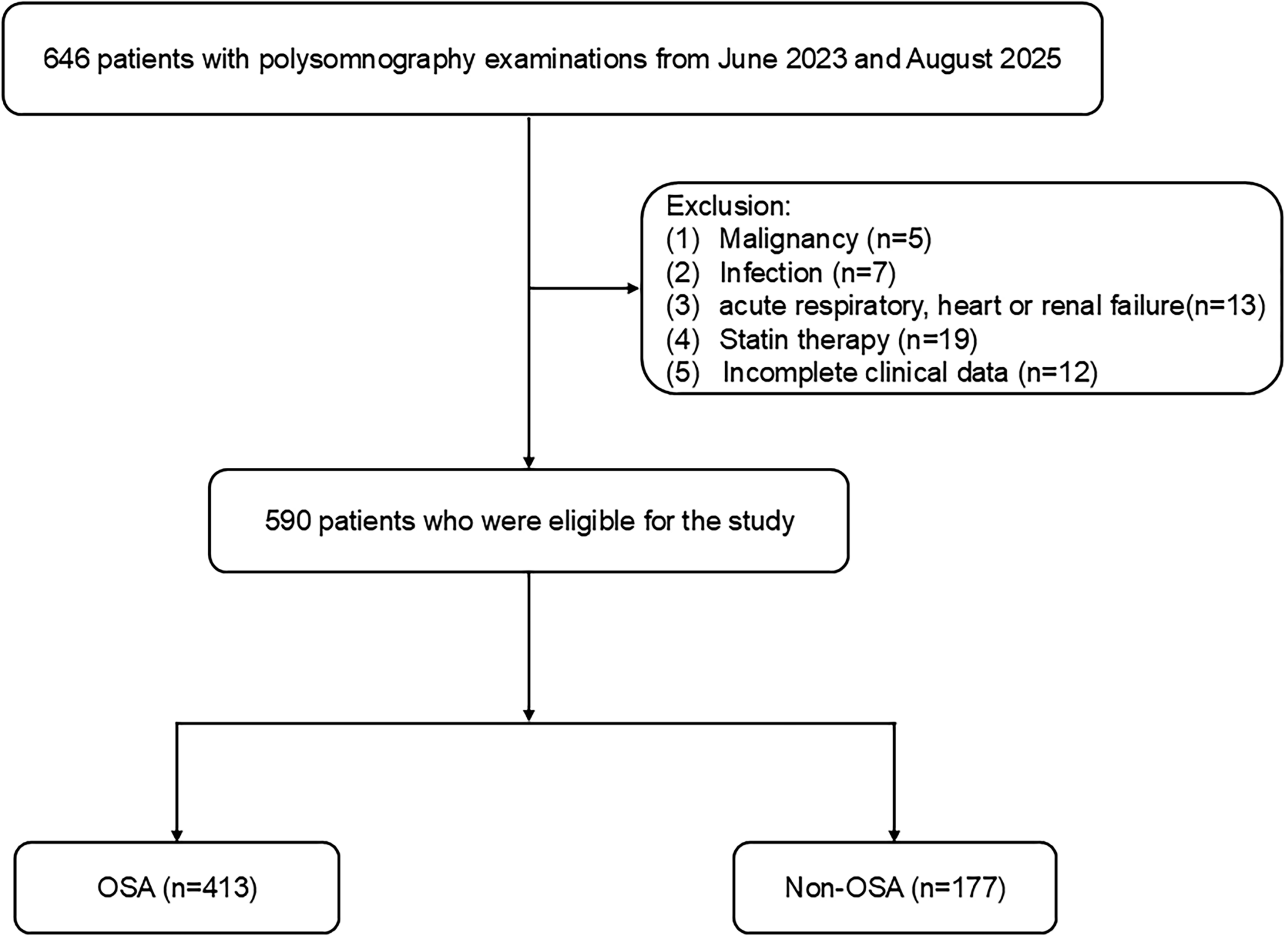
Flow chart of participant enrollment. OSA, obstructive sleep apnea.

Between June 2023 and August 2025, consecutive patients presenting with suspected OSA were retrospectively recruited from Changsha Central Hospital. The study protocol was approved by the Ethics Committee of Changsha Central Hospital. Inclusion criteria consisted of: (1) the presence of at least one clinical symptom indicative of OSA, such as snoring, excessive daytime sleepiness, witnessed nocturnal choking, or apnea; (2) no prior diagnosis of OSA or history of OSA-related treatments, including upper airway surgery, mandibular advancement devices, or positive airway pressure therapy (e.g., CPAP or Auto-CPAP); and (3) age ≥18 years with a minimum recorded sleep time of 4 h during polysomnography. Exclusion criteria were: (1) history of malignancy within the past 10 years; (2) acute infection within the preceding 2 months; (3) diagnosed autoimmune disorders; (4) acute cardiac events or respiratory failure; and (5) presence of nasal polyps. All participants underwent overnight, attended, in-laboratory polysomnography using the iRem-A system (Physio Med, Hangzhou, China) according to a standardized institutional protocol. Signals were acquired through surface electrodes for electroencephalography (EEG: F4-M1, C4-M1, O2-M1), electrocardiography (ECG), electrooculography (EOG: left and right outer canthi), and submental electromyography (EMG). Additional recordings included oronasal airflow (using a combined thermistor and nasal pressure transducer), thoracic and abdominal respiratory effort (via respiratory inductance plethysmography belts), tracheal sound (via microphone), and continuous transcutaneous peripheral oxygen saturation (SpO₂) via pulse oximetry. Body position during sleep was also documented by a position sensor. The recording commenced at the patient’s habitual bedtime and continued until spontaneous awakening the next morning, aiming for a minimum of 7 h in bed. Data acquisition was performed using a computerized polysomnography system (iRem-A software, version 3.2). All polysomnography recordings were scored blinded by a certified sleep technician. In addition, the technician was unaware of the clinical characteristics of the patients, laboratory results (including complete blood counts, SII, and SIRI), and study hypotheses. A total of 590 patients were included in the final analysis. The patient enrollment process is detailed in Figure 1.

## Clinical assessment and subject grouping

Sleep stages were scored according to the standardized criteria of Rechtschaffen and Kales. Respiratory events were identified based on the American Academy of Sleep Medicine (AASM) guidelines. Apnea was defined as a ≥90% reduction in airflow from baseline lasting ≥10 s. Hypopnea was defined as a ≥30% decrease in airflow for ≥10 s, accompanied by either a ≥3% drop in oxygen saturation or an arousal. The apnea–hypopnea index (AHI) was calculated as the total number of apneas and hypopneas per hour of sleep. OSA diagnosis was established according to the International Classification of Sleep Disorders, Third Edition (ICSD-3), requiring either: AHI ≥ 5 events/h with associated daytime or nocturnal symptoms or comorbidities, or AHI ≥ 15 events/h irrespective of symptoms. Patients diagnosed with OSA were further stratified by severity as follows: mild (AHI 5–15), moderate (AHI 16–30), and severe (AHI > 30).

## Data collection

Participant data encompassing demographics, clinical characteristics, and polysomnographic parameters—namely age, sex, body mass index (BMI), current smoking and drinking habits, histories of diabetes, coronary artery disease, hypertension, and atrial fibrillation, along with lowest SpO₂, sleep latency, and sleep efficiency—were systematically recorded. BMI was calculated as weight in kilograms divided by height in meters squared (kg/m^2^). Hypertension was established based on a systolic/diastolic blood pressure ≥140/90 mmHg, use of antihypertensive therapy, or a documented medical history. Diabetes mellitus was defined as a fasting glucose level ≥126 mg/dL, use of glucose-lowering agents, or a previous diagnosis. A diagnosis of atrial fibrillation required either electrocardiographic documentation of irregular atrial activity or a positive clinical history. Coronary artery disease was defined as a prior myocardial infarction, prior coronary revascularization, angiographic evidence of significant coronary stenosis, or a previously established diagnosis. Current smoking was defined as consumption of at least 10 cigarettes per day for a minimum of 5 years prior to enrollment. Similarly, current drinking was defined as regular intake of at least 20 grams of ethanol per day for at least 5 years.

After a minimum 8-h fasting period, venous blood specimens were collected from 6:00 to 7:00 a.m. Complete blood counts for white blood cells (WBC), neutrophils (N), monocytes (M), platelets (P), and lymphocytes (L) were subsequently measured using an automated hematology analyzer (BZ6800, China). The systemic immune-inflammation index (SII) and systemic inflammation response index (SIRI) were calculated as SII = P × (N/L) and SIRI = N × (M/L), respectively, based on the measured cellular counts (16).

### Statistical analysis

Statistical analyses were conducted using SPSS version 25.0 (IBM Corp., Armonk, NY, United States) and MedCalc version 15.6.0 (MedCalc Software, Ostend, Belgium). Normality of continuous variables was evaluated via the Kolmogorov–Smirnov test. Data following a normal distribution are shown as mean ± SD; non-normally distributed data are presented as median (IQR). Categorical variables are summarized as n (%). Between-group differences were assessed using the Chi-square or Fisher’s exact test for categorical variables, and the Student’s *t*-test or Mann–Whitney U test for continuous variables, depending on distribution. Multicollinearity was examined using standard collinearity diagnostics. Box plots were generated to illustrate SII and SIRI levels across OSA severity groups. Correlations between SII/SIRI and AHI or lowest SpO₂ were examined by Spearman’s rank correlation. Binary logistic regression analysis was conducted to determine factors independently associated with OSA, with additional subgroup and interaction analyses. The diagnostic performance of SII and SIRI for identifying OSA was assessed using receiver operating characteristic (ROC) curve analysis. For the individual indices (SII and SIRI), ROC curves were generated using their raw, continuous laboratory values. To evaluate the combined predictive value of SII and SIRI, we constructed a binary logistic regression model with OSA status (yes/no) as the dependent variable. The independent variables for this primary combined model were SII and SIRI, both entered as continuous predictors. The rationale for selecting only SII and SIRI in this primary model was to evaluate the intrinsic and unconfounded discriminatory ability of these novel hematological indices themselves, which is a standard approach for initial biomarker assessment. The predicted probabilities (p) generated from this logistic regression model (*p* = 1 / [1 + e^-(β0 + β1*SII + β2*SIRI)]) were then used to generate the ROC curve for the ‘SII + SIRI’ combination. The area under the curve (AUC) with its 95% confidence interval (CI) was calculated for each ROC curve using the non-parametric method. The optimal cut-off point for each predictor was determined by maximizing the Youden Index (J = Sensitivity + Specificity − 1). To statistically compare the discriminatory ability of the combined model against each index alone, the DeLong test for two correlated ROC curves was employed. Moreover, we have conducted a multiclass ROC analysis using R 4.3 (R Foundation for Statistical Computing, Vienna, Austria) to evaluate the predictive performance of the combined SII/SIRI model across OSA severity categories. All tests were two-tailed, and a *p*-value < 0.05 was considered statistically significant.

## Results

### Clinical and demographic characteristics of patients with OSA and non-OSA

**Figure 2:**
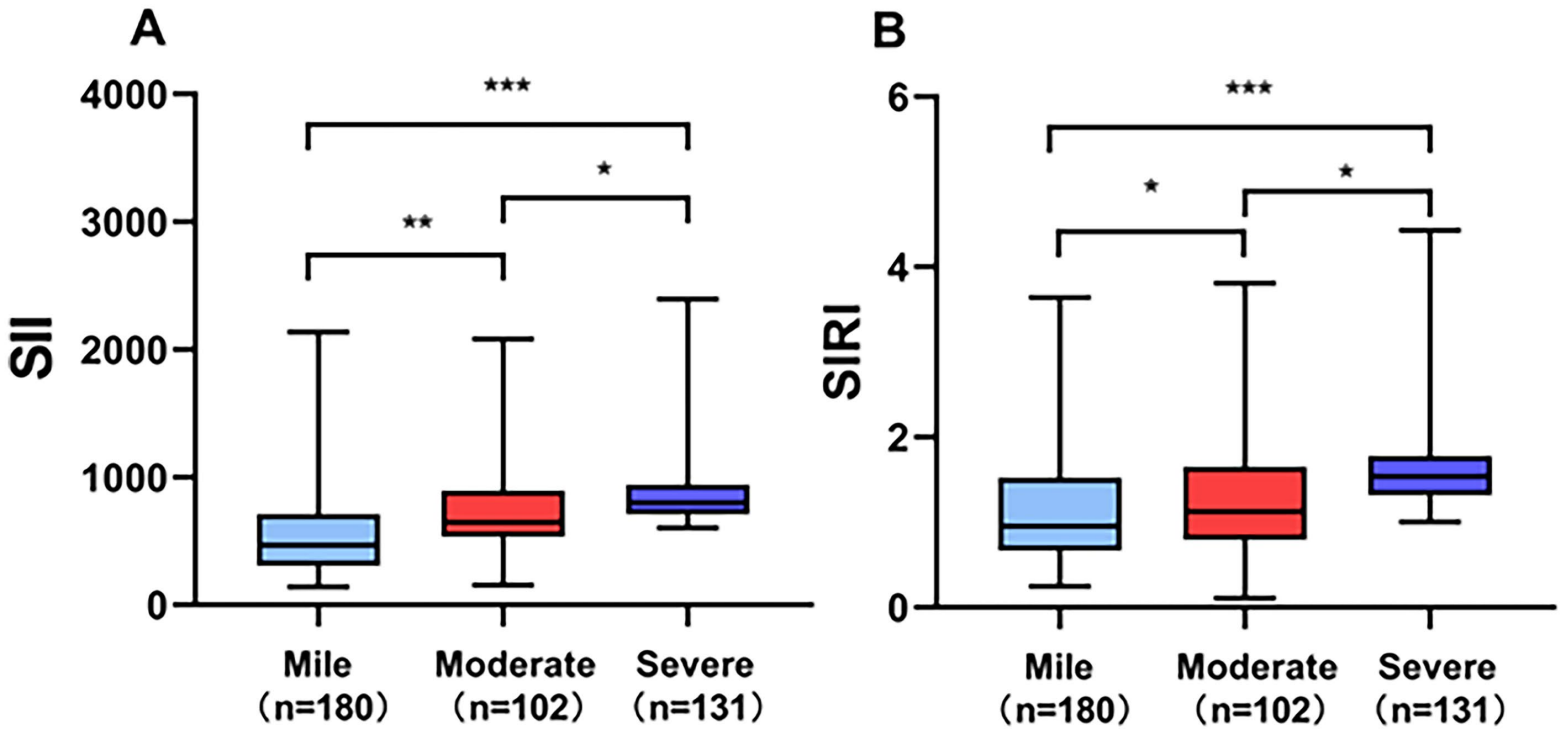
Comparison of **(A)** SII and **(B)** SIRI across OSA severity groups. ***p < 0.001, **p < 0.01, *p < 0.05.

Table 1 summarizes the baseline characteristics of all enrolled participants. Among the 590 patients included in the study, 413 (70%) were diagnosed with OSA, while 177 (30%) comprised the non-OSA group. Compared to the non-OSA group, patients with OSA showed significantly higher proportions of males (*p* = 0.006), as well as significantly elevated values of BMI (*p* < 0.001), prevalence of hypertension (*p* = 0.002), AHI (*p* < 0.001), SII (*p* < 0.001), and SIRI (*p* < 0.001). Conversely, the lowest SpO₂ was significantly reduced in the OSA group (*p* < 0.001). Additionally, as illustrated in Figure 2, both SII and SIRI values increased significantly with OSA severity.

**Table 1:** Baseline characteristics.

Variable	Total (*n* = 590)	Non-OSA (*n* = 177)	OSA (*n* = 413)	*P*
Age, years	57.94 ± 15.44	54.84 ± 17.67	59.27 ± 14.20	0.001
Male, *n* (%)	438 (74.24)	118 (66.67)	320 (77.48)	0.006
BMI, kg/m^2^	25.40 ± 4.92	23.58 ± 4.51	26.19 ± 4.89	<0.001
Current smoking	242 (41.02)	69 (38.98)	173 (41.89)	0.511
Current drinking	120 (20.34)	33 (18.64)	87 (21.07)	0.503
Hypertension	372 (63.05)	95 (53.67)	277 (67.07)	0.002
Diabetes mellitus	151 (25.60)	38 (21.47)	113 (27.36)	0.133
Atrial fibrillation	94 (15.93)	26 (14.69)	68 (16.46)	0.589
Coronary artery disease	121 (20.51)	31 (17.51)	90 (21.79)	0.238
Lowest SpO2 (%)	84 (79–88)	88 (85–91)	82 (77–86)	<0.001
AHI (/h)	11.44 (3.43–25.76)	1.37 (0.43–2.94)	17.69 (10.47–36.51)	<0.001
WBC (×10^9^/L)	6.91 (5.83–8.19)	6.89 (5.87–8.12)	6.91 (5.82–8.19)	0.860
Neutrophils (×10^9^/L)	4.37 (3.50–5.42)	4.33 (3.51–5.43)	4.39 (3.47–5.42)	0.685
Monocytes	0.40 (0.30–0.50)	0.38 (0.30–0.47)	0.40 (0.30–0.50)	0.071
Platelets	203 (168–243)	195 (161.50–235.50)	207 (170–246)	0.132
Lymphocytes	1.70 (1.30–2.34)	1.71 (1.28–2.39)	1.70 (1.30–2.35)	0.615
SII	534 (364.24–753.96)	388 (285.37–482.56)	623 (463.74–817.85)	<0.001
SIRI	1.11 (0.73–1.55)	0.91 (0.61–1.27)	1.24 (0.79–1.65)	<0.001

BMI, body mass index; AHI, Apnea-hypopnea index; WBC, white blood cell; SII, systemic inflammation index; SIRI, systemic immune-inflammatory index.

### Correlation analysis of SII, SIRI, AHI, and lowest SpO₂ in patients with OSA

**Figure 3:**
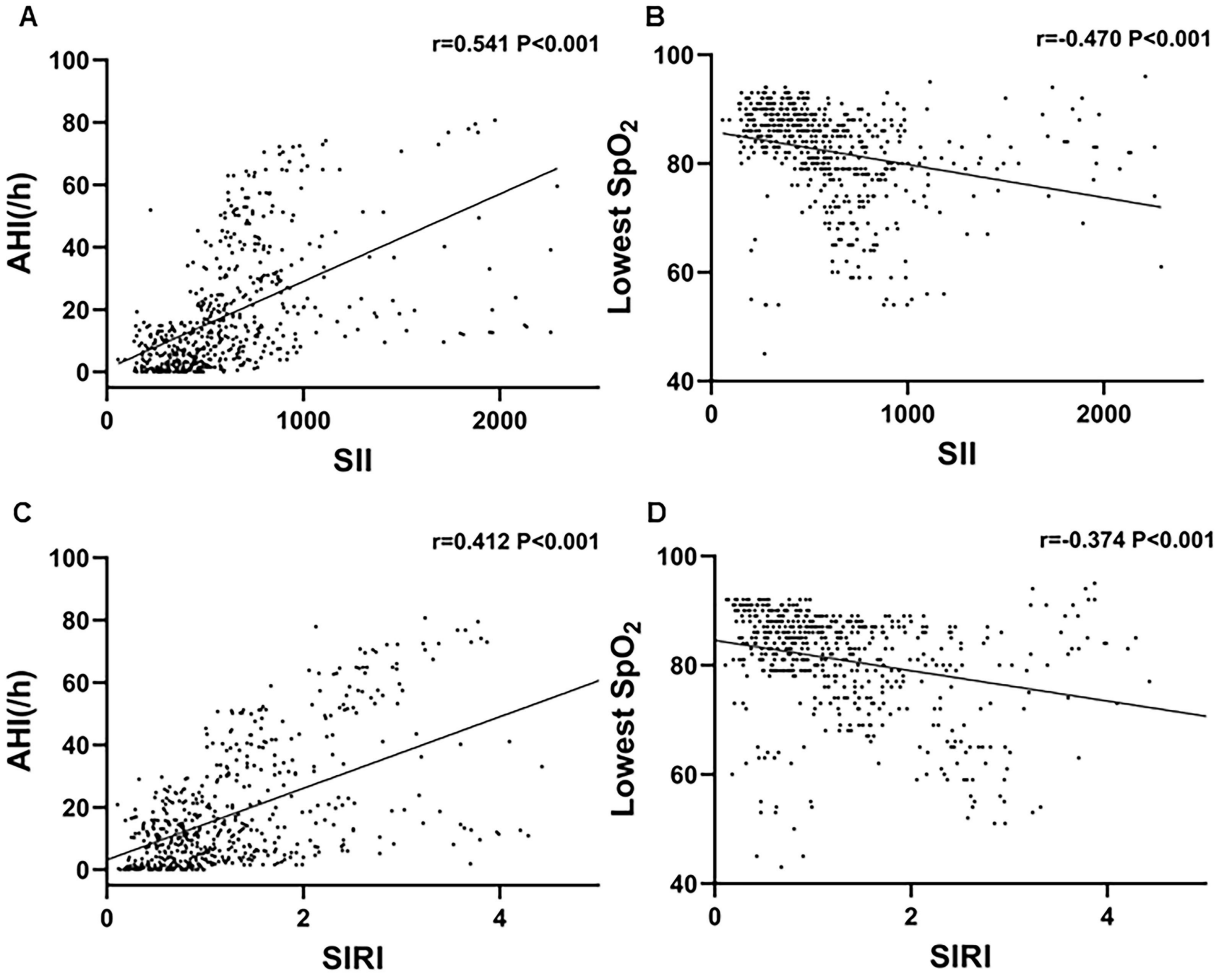
Scatter plots demonstrating the correlations of SII and SIRI with AHI and lowest SpO_2_. **(A)** SII was positively correlated with AHI (*r* = 0.541, *p* < 0.001). **(B)** SII was negatively correlated with lowest SpO (*r* = −0.470, *p* < 0.001). **(C)** SIRI was positively correlated with AHI (*r* = 0.412, *p* < 0.001). **(D)** SIRI was negatively correlated with lowest SpO (*r* = −0.374, *p* < 0.001).

Spearman’s rank correlation analysis was performed to evaluate the relationships among SII, SIRI, AHI, and the lowest SpO₂. A strong positive correlation was observed between SII and AHI (*r* = 0.541, *p* < 0.001), as well as a moderate positive correlation between SIRI and AHI (*r* = 0.412, *p* < 0.001). Conversely, both SII (*r* = −0.470, *p* < 0.001) and SIRI (*r* = −0.374, *p* < 0.001) were negatively correlated with the lowest SpO₂ (Figure 3).

### Subgroup analyses and interaction test

**Figure 4:**
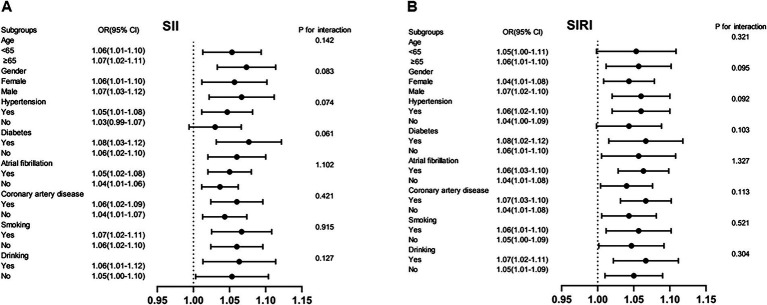
Subgroup analyses of **(A)** SII, **(B)** SIRI, and OSA.

Stratified analyses were conducted to assess the consistency of the associations between SII, SIRI, and OSA across prespecified demographic and clinical subgroups (Figure 4). The positive associations of both SII and SIRI with OSA remained consistent in subgroups stratified by age (<65 vs. ≥65 years), gender (female vs. male), diabetes mellitus, hypertension, coronary artery disease, atrial fibrillation, smoking status, and alcohol consumption. No significant interaction effects were observed in any subgroup (all *P* for interaction > 0.05). These findings demonstrate the robustness and broad generalizability of our conclusions across diverse population subgroups.

### Logistic regression analysis of OSA risk factors

Table 2 summarizes the results of the rudimentary OSA models. Based on the results of Table 1, variables with significant between-group differences were fitted into a multivariate binary logistic regression model to determine independent predictors of OSA. No significant collinearity was detected, as indicated by variance inflation factors (VIF) of 1.32 for SII and 2.91 for SIRI. The analysis identified the following independent risk factors for OSA: male (OR = 1.301; 95% CI: 1.142–1.537; *p* = 0.006), BMI (OR = 1.283; 95% CI: 1.083–1.631; *p* = 0.002), SII (OR = 1.601; 95% CI: 1.352–1.987; *p* < 0.001), and SIRI (OR = 1.459; 95% CI: 1.182–1.750; *p* < 0.001). Furthermore, SII and SIRI were analyzed as categorical variables based on tertiles. Following the adjustment for confounding variables, patients exhibiting elevated SII levels (3rd quartile vs. 1st quartile, OR, 1.852; 95% CI, 1.308–2.316, *p* = 0.004) and SIRI levels (3rd quartile vs. 1st quartile, OR, 1.681, 95% CI, 1.292–2.410, *p* = 0.009) exhibited a significantly increased risk of OSA (Table 3).

**Table 2:** Logistic regression analysis for risk factors associated with OSA.

Variable	OR (95% CI)	*P*	Adjusted OR (95% CI)	*P*
Age	1.309 (1.207–1.503)	0.002	1.284 (1.028–1.400)	0.274
Male	1.643 (1.362–1.982)	<0.001	1.301 (1.142–1.537)	0.006
BMI	1.405 (1.243–1.783)	<0.001	1.283 (1.083–1.631)	0.002
Hypertension	1.211 (1.142–1.654)	0.003	1.104 (1.009–1.461)	0.315
SII	1.915 (1.672–2.407)	<0.001	1.601 (1.352–1.987)	<0.001
SIRI	1.875 (1.340–2.387)	<0.001	1.459 (1.182–1.750)	<0.001

**Table 3:** Association between SII, SIRI, and OSA.

Variable	OR (95% CI)	*P*	Adjusted OR (95% CI)^a^	*P*
SII ternary classification				
T1	Reference		Reference	
T2	1.551 (1.293–2.241)	<0.001	1.312 (1.009–1.873)	0.041
T3	2.253 (1.714–2.763)	<0.001	1.852 (1.308–2.361)	0.004
SIRI ternary classification				
T1	Reference		Reference	
T2	1.493 (1.241–1.889)	<0.001	1.300 (1.021–1.907)	0.032
T3	2.184 (1.702–2.853)	<0.001	1.681 (1.292–2.410)	0.009

SII: T1 ≤ 425.84; 425.84 < T2 ≤ 663.90; T3 > 663.90. SIRI: T1 ≤ 0.85; 0.85 < T2 ≤ 1.40; T3 > 1.40.

a Model: adjusted for age, Male, BMI, and Hypertension.

### ROC curve analysis of diagnostic performance for OSA

**Figure 5:**
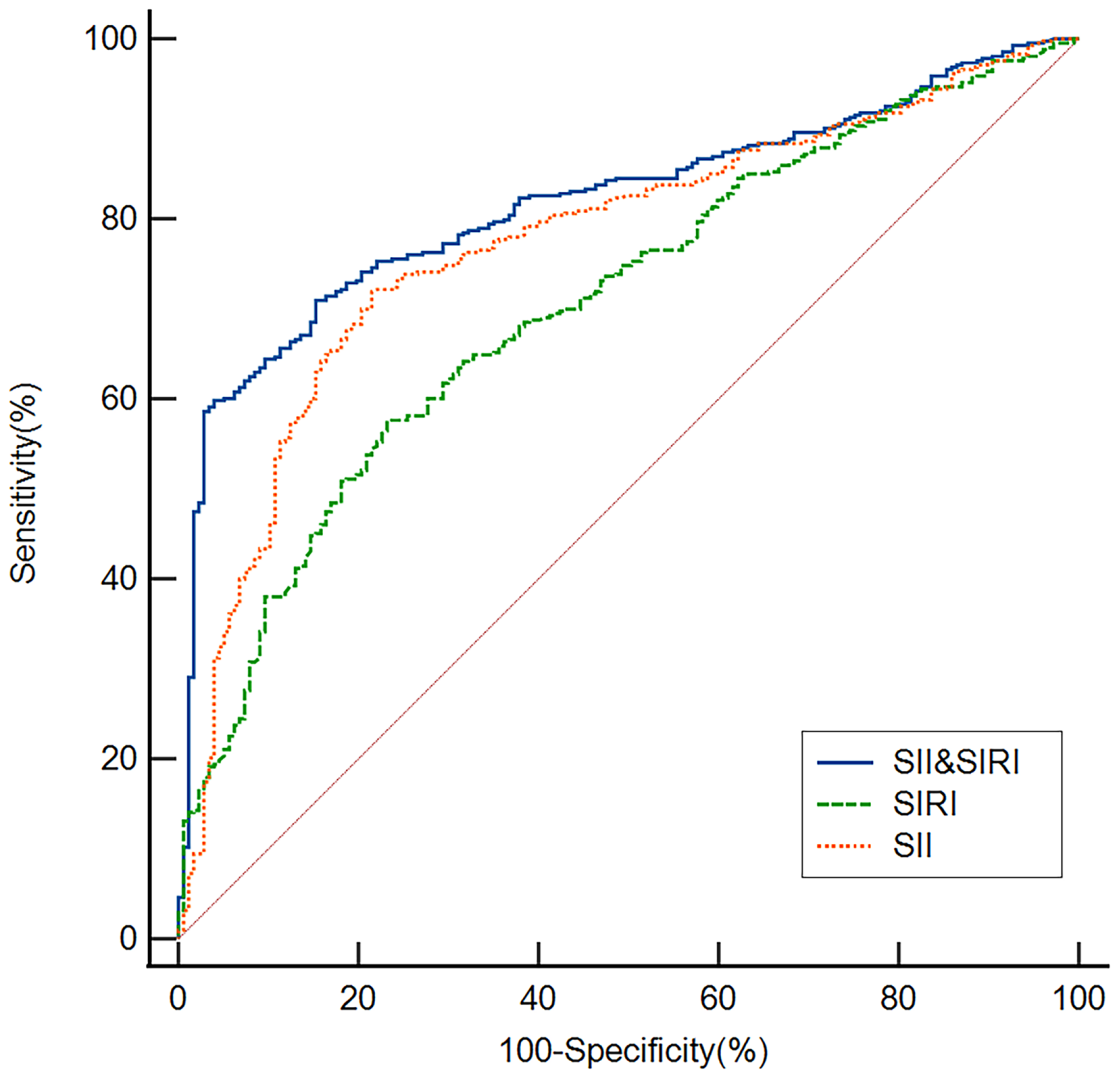
ROC curves demonstrating the discriminatory power of the SII, SIRI, and their combination in identifying patients with OSA. The AUC were 0.774 for SII, 0.705 for SIRI, and 0.819 for the combined model.

As shown in Figure 5, ROC curve analysis was performed to evaluate the discriminatory ability of SII and SIRI in identifying patients with OSA. The area under the curve (AUC) for SII was 0.774 (95% CI: 0.738–0.807; *p* < 0.001), with an optimal cutoff value of 493, yielding a sensitivity of 71.91% and a specificity of 78.53%. For SIRI, the AUC was 0.705 (95% CI: 0.666–0.742; *p* < 0.001), with a cutoff value of 1.21, corresponding to a sensitivity of 57.63% and a specificity of 76.84%. Furthermore, the combination of SII and SIRI was assessed for its diagnostic performance. The combined model achieved an AUC of 0.819 (95% CI: 0.786–0.849; *p* < 0.001), with a cutoff value of 0.75, a sensitivity of 59.81%, and a specificity of 86.05%. These results indicate that both SII and SIRI possess significant discriminatory power for OSA, with their combination further enhancing diagnostic accuracy.

### Performance of the combined model in differentiating OSA severity levels

To further quantify the discriminative ability across multiple classes, we performed a multiclass ROC analysis. The combined model achieved an overall multiclass AUC (estimated by the Hand & Till method) of 0.762 (95% CI: 0.728–0.795) for distinguishing between the four severity categories. The pairwise AUCs for discriminating adjacent severity levels were: Non-OSA vs. Mild OSA = 0.781 (95% CI: 0.738–0.824, *p* < 0.001), Mild vs. Moderate OSA = 0.658 (95% CI: 0.602–0.714, *p* < 0.001), and Moderate vs. Severe OSA = 0.672 (95% CI: 0.617–0.727, *p* < 0.001).

## Discussion

To our knowledge, this is the first study to concurrently evaluate the associations of both the SII and SIRI with OSA, especially in an Asian population. Our findings yield several novel insights. First, both SII and SIRI were significantly elevated in patients with OSA compared to non-OSA controls. Moreover, these indices showed a significant positive correlation with the AHI and a negative correlation with the lowest SpO₂. Second, binary logistic regression identified SII and SIRI as independent risk factors for OSA. Furthermore, a sensitivity analysis that further adjusted for additional comorbid conditions (atrial fibrillation, diabetes mellitus, coronary artery disease,) and lifestyle factors (alcohol consumption, smoking) confirmed the robustness of these associations (all *p* > 0.05). This strengthens the argument that the relationship between these novel inflammatory indices and OSA is not merely mediated by these common confounders. Finally, ROC analysis indicated that the combination of SII and SIRI has strong discriminatory power for OSA. Together, these results underscore the significant roles of SII and SIRI as reliable inflammatory biomarkers associated with OSA.

The SII, which integrates platelet, neutrophil, and lymphocyte counts, serves as a composite marker reflecting both systemic inflammation and immune response. It is not only cost-effective and stable but also highly reproducible, making it a practical tool for clinical and research applications (34). A retrospective cohort study reported a positive correlation between SII and the prevalence of OSA, with SII levels increasing alongside disease severity (21). Analysis of NHANES data further revealed a strong link between SII and sleep disturbances, suggesting that inflammatory markers such as SII may mediate the association between sedentary behavior and sleep disorders (35). Given the complexity and expense of PSG, recent studies have proposed the use of accessible hematological markers like SII to evaluate the effectiveness of sleep surgery (36). Additionally, a study involving U. S. adults identified positive correlations between SII and sleep problems, OSA symptoms, and daytime sleepiness (23). Consistent with these reports, our study found significantly higher SII levels in OSA patients compared to non-OSA controls. We also observed a positive correlation between SII and AHI, and a negative correlation with the lowest SpO₂. Binary logistic regression confirmed SII as an independent risk factor for OSA. These findings align with previous literature indicating elevated SII in OSA populations. Nonetheless, the relationship between SII and OSA remains controversial. Some studies have reported a significant correlation only in severe OSA subgroups (24), while others found no association with disease severity (25). To date, evidence regarding SII and sleep disorders remains limited and derived predominantly from specific populations. Thus, our study contributes valuable insights to this evolving area of research.

The SIRI is a novel integrated biomarker that reflects systemic inflammatory status and immune response. Growing evidence has associated SIRI with prognosis in various diseases, including pneumonia, rheumatoid arthritis, and acute pancreatitis (26–28). It has also been applied in cardiovascular risk stratification, demonstrating high predictive value for incident cardiovascular diseases (29–32). A meta-analysis indicated that sleep disturbances are correlated with elevated systemic inflammatory markers such as CRP (37). Furthermore, disrupted rest-activity circadian rhythms have been linked to increased leukocyte-based inflammatory indices (38). Concurrently, systemic inflammation—whether from endogenous triggers or infections—can disrupt normal sleep architecture via cytokine-mediated communication across the blood–brain barrier, influencing central nervous system functions through both neural and humoral pathways (10). Previous research has shown that proinflammatory cytokines released by TLR4-stimulated monocytes at bedtime can predict sleep efficiency and slow-wave sleep duration (39). Although the clinical relevance of SIRI is increasingly recognized across disciplines, its association with OSA remains underexplored. To our knowledge, this is the first study to systematically evaluate the predictive utility of SIRI for OSA. Our results demonstrated significantly higher SIRI levels in patients with OSA compared to non-OSA controls. SIRI was positively correlated with AHI and negatively correlated with the lowest SpO₂. Binary logistic regression confirmed SIRI as an independent risk factor for OSA. These findings broaden the understanding of SIRI’s role in OSA and may provide new insights for future therapeutic strategies.

Inflammatory cytokines such as interleukins and tumor necrosis factor (TNF) are known to play key roles in mediating inflammation and sleep regulation (14, 40). These signaling molecules exhibit dysregulation in OSA (41) and other sleep disorders (42). Sleep, governed by the central nervous system, dynamically modulates immune function through the production and redistribution of inflammatory cytokines (10). Experimental sleep deprivation disrupts the circadian rhythm of IL-6 secretion, reducing nocturnal release while increasing daytime levels, and similarly dysregulates TNF production (43, 44). A meta-analysis further supports that prolonged sleep duration and sleep disorders were associated with elevated CRP and IL-6 levels (37). Notably, treating sleep disorders can normalize inflammatory markers, thereby attenuating systemic inflammation (45). These findings suggest that sleep disturbances may compromise host health through sustained inflammatory activation (46). Conversely, immune imbalance may also impair sleep quality; for instance, influenza-infected mice exhibit increased non-REM sleep and reduced REM sleep (47), and septic rats show sleep fragmentation linked to elevated inflammatory cytokines (48).

Our findings, which position SII and SIRI as robust biomarkers for OSA, invite an exploration of the biological pathways underlying this association. We propose several plausible explanatory mechanisms. The components of these indices—neutrophils, lymphocytes, monocytes, and platelets—are central players in systemic inflammation, and their dysregulation in OSA may be both a consequence and a contributor to disease progression through several interrelated pathways. First, intermittent hypoxia (IH), a hallmark of OSA, is a potent activator of innate immunity (49, 50). It induces a state of oxidative stress and promotes the release of pro-inflammatory cytokines such as IL-6 and TNF-α, which in turn stimulate neutrophilia and monocytosis—key numerators in SII and SIRI (51). Second, the relative lymphopenia observed in systemic inflammatory states (reflected in the denominator of both indices) may result from glucocorticoid-mediated apoptosis or margination of lymphocytes (52). This lymphopenia could signify a shift toward a pro-inflammatory state and an impaired adaptive immune response, possibly reducing the capacity for tissue repair and homeostasis in the respiratory tract. Third, monocytes, elevated in SIRI, are precursors to tissue macrophages. In the context of OSA, IH and sleep fragmentation can promote the polarization of macrophages toward a pro-inflammatory (M1) phenotype in adipose tissue and the vascular endothelium, driving insulin resistance and endothelial dysfunction—conditions that are both comorbidities and potential amplifiers of OSA severity (53, 54). A recent study showed that IH leads to the polarization of M1-type macrophages through NSUN6-mediated ferroptosis, leading to adipose tissue inflammation-a key pathway between OSA and metabolic dysfunction (55). Finally, the platelet count, a component of SII, is particularly relevant. IH can trigger thrombopoiesis and platelet activation (56). A previous study identified a novel mechanism: acoustic vibration associated with snoring itself can mechanically activate platelets independent of biochemical pathways, suggesting that the mechanical forces of snoring may directly contribute to the prothrombotic state of OSA (57). Beyond their role in coagulation, activated platelets are potent inflammatory modulators, releasing cytokines and forming aggregates with leukocytes (platelet-leukocyte complexes), thereby amplifying the systemic inflammatory cascade and contributing to the heightened cardiovascular risk observed in OSA patients (56, 58). Therefore, SII and SIRI likely serve as integrative biomarkers, capturing the multifaceted inflammatory-immune-thrombotic cross-talk that is upregulated in OSA (59). They reflect not merely the presence of inflammation, but a specific dysregulation of cellular immune components driven by IH and sleep fragmentation, which may perpetuate a vicious cycle of upper airway dysfunction, metabolic dysregulation, and end-organ damage. Future mechanistic studies are warranted to directly correlate these indices with *in vivo* measures of upper airway inflammation, detailed hypoxia patterns, and specific immune cell functional assays to validate these proposed pathways.

Furthermore, our study identified BMI and male sex as independent factors associated with OSA, which is consistent with previous reports (7). OSA is more prevalent in males, although postmenopausal females exhibit a significantly increased incidence, likely due to decreased estrogen levels (60). Our results also align with existing literature indicating that obesity is an independent risk factor for OSA (61–63). While older age has been previously implicated as a risk factor for OSA (7), our analysis did not find a significant association between age and OSA in this study. We suggest that discrepancies among studies may be attributed to differences in the ethnic background of study populations, sample sizes, and variations in disease severity.

Given the significant heterogeneity in OSA, it is unlikely that any single biomarker can accurately predict its risk or progression. Therefore, integrating multiple biomarkers is essential to improve OSA risk estimation. In this study, we used ROC curves to evaluate the combined discriminatory ability of the SII and SIRI in distinguishing patients with OSA. Our findings indicate that both SII and SIRI effectively differentiate OSA from non-OSA cases, with SII demonstrating superior discriminatory power compared to SIRI. Notably, the combination of SII and SIRI achieved an AUC of 0.819, significantly enhancing the predictive capability for OSA compared to either marker alone. Furthermore, the multiclass ROC analysis further indicated that the model performs best in discriminating between the absence of OSA and mild disease (AUC 0.781), with more modest performance in differentiating between adjacent severity levels among established OSA patients (e.g., Mild vs. Moderate AUC 0.658). This pattern suggests that while systemic inflammation, as captured by these indices, rises significantly with the onset of OSA, the incremental increase per unit of AHI gain within the moderate-to-severe range may be less pronounced or more variable. This underscores the potential of these indices as screening tools to flag significant disease, while also highlighting that other factors (e.g., anatomical factors) may play an important role in determining precise severity stratification among patients with established OSA.

Our findings position SII and SIRI not as replacements for PSG but as accessible, low-cost adjuncts for OSA risk stratification and screening. These indices could be integrated into clinical practice in several ways: in primary care, elevated SII/SIRI in symptomatic or high-risk patients could prompt prioritized referral for definitive sleep testing; in sleep clinics, they may offer preliminary insight into inflammatory burden and potential severity while patients await PSG; and future studies could explore their role in monitoring treatment response. Their primary value lies in complementing existing tools by optimizing resource allocation, especially in settings with limited access to PSG. However, key challenges must be addressed before widespread adoption, including the non-specific nature of systemic inflammation, the need for validation of optimal cut-off values across diverse populations, the development of practical tools for seamless clinical workflow integration, and formal cost-effectiveness analyses to demonstrate systemic benefits. Future research should focus on prospective validation and implementation studies to define their optimal use within multi-level healthcare systems.

This study has several limitations: (1) As a cross-sectional study conducted exclusively in a Chinese population, the findings may introduce inherent selection biases and require validation in non-Chinese cohorts. Future large-scale longitudinal studies are needed to confirm these results; (2) the predominance of male participants may introduce gender-related bias; (3) given that SII and SIRI are derived from neutrophil, monocyte, platelet, and lymphocyte counts—each of which may independently influence OSA risk—the composite indices should be interpreted cautiously. Further multi-center studies with larger sample sizes are warranted to verify their clinical utility; (4) potential confounding factors, such as medication use, physical activity, diet, subclinical inflammation from other sources, were not fully adjusted for in the analysis; (5) future investigations should incorporate additional circulating inflammatory biomarkers to improve risk stratification and clarify causal mechanisms; (6) the lack of data on other established inflammatory biomarkers (e.g., hs-CRP, IL-6) precludes a direct comparison of the predictive performance of SII and SIRI against these markers. In future studies, we will verify the superiority of the combined use of SII and SIRI in predicting obstructive sleep apnea, compared with other inflammatory markers; and (7) as this study enrolled only individuals with high clinical suspicion of OSA, the generalizability of the results to the broader population should be approached with caution.

## Conclusion

In conclusion, our study demonstrates that both the SII and SIRI serve as independent risk factors for OSA and represent readily accessible, cost-effective biomarkers with potential utility in OSA screening. Moreover, the combination of SII and SIRI exhibits enhanced predictive value compared to either marker alone, suggesting that integrated inflammatory indices may improve risk stratification. Nonetheless, further validation through prospective multi-center studies and mechanistic research is essential to confirm these findings and elucidate the underlying pathophysiology of inflammation in OSA.

## Data Availability

The raw data supporting the conclusions of this article will be made available by the authors, without undue reservation.
